# A Coupled Inflammatory–Fibrotic–Nitrosative Axis in Patients with Patent Foramen Ovale and Cryptogenic Stroke

**DOI:** 10.3390/jcm15114256

**Published:** 2026-05-31

**Authors:** Michał Tworek, Katarzyna Trojanowicz, Zuzanna Sachajko, Monika Kowalik-Pandyra, Klaudia Pacia, Klaudia Bielecka, Sylwia Szczepara, Miłosz Tworek, Joanna Natorska, Grzegorz Kopeć, Monika Komar

**Affiliations:** 1 Department of Cardiac and Vascular Diseases, Krakow Specialist Hospital Named After St. John Paul II, ul. Prądnicka 80, 31-202 Krakow, Poland; k.trojanowicz@szpitaljp2.krakow.pl (K.T.); zuzannasachajko@doctoral.uj.edu.pl (Z.S.); monika.kowalik01@gmail.com (M.K.-P.); klaudia.knap87@gmail.com (K.P.); kl.bbielecka@gmail.com (K.B.); sszczepara@gmail.com (S.S.); grzegorzkrakow1@gmail.com (G.K.); moni_s@interia.pl (M.K.); 2 Department of Cardiac and Vascular Diseases, Institute of Cardiology, Jagiellonian University Medical College, Krakow Specialist Hospital Named After St. John Paul II, ul. Prądnicka 80, 31-202 Krakow, Poland; 3 Doctoral School of Medical and Health Sciences, Jagiellonian University Medical College, ul. Św. Łazarza 16, 31-530 Krakow, Poland; 4 The University Hospital in Krakow, ul. Marii Orwid 11, 30-688 Krakow, Poland; milosz.tworek.med@gmail.com; 5 Department of Thromboembolic Disorders, Institute of Cardiology, Jagiellonian University Medical College, Krakow Specialist Hospital Named After St. John Paul II, ul. Prądnicka 80, 31-202 Krakow, Poland; jnatorska@szpitaljp2.krakow.pl; 6 Krakow Centre for Medical Research and Technologies, Krakow Specialist Hospital Named After St. John Paul II, ul. Prądnicka 80, 31-202 Krakow, Poland

**Keywords:** patent foramen ovale, cryptogenic stroke, inflammation, nitrosative stress, endothelial dysfunction, biomarkers

## Abstract

**Background:** Patent foramen ovale (PFO) is associated with cryptogenic stroke, but mechanisms beyond paradoxical embolism remain unclear. Inflammation, fibrosis-related remodeling, and nitrosative stress may contribute to vascular vulnerability in these patients. The aim of this study was to evaluate inflammatory, fibrotic, and nitrosative biomarker profiles in patients with PFO and prior cryptogenic stroke and to determine whether these patients exhibit coupled inflammatory fibrotic nitrosative activation. **Methods:** This prospective, observational, single-center study included 92 patients aged <55 years with PFO and prior cryptogenic stroke and 56 age-matched controls without PFO and cerebrovascular events. Circulating interleukin-18 (IL-18), galectin-3, and 3-nitrotyrosine (3-NT) levels were measured using ELISA assays. Correlation and linear regression analyses were performed. **Results:** Compared with controls, patients with PFO had higher galectin-3 (11.87 vs. 10.36 ng/mL; *p* = 0.015), IL-18 (268.0 vs. 121.0 pg/mL; *p* < 0.001), and 3-NT (48.5 vs. 41.8 ng/mL; *p* = 0.046). Significant correlations were observed between IL-18 and galectin-3 (r = 0.565), IL-18 and 3-NT (r = 0.425), and galectin-3 and 3-NT (r = 0.292) (all *p* ≤ 0.002). **Conclusions:** Patients with PFO and prior cryptogenic stroke exhibit a distinct biomarker profile consistent with coupled inflammatory–fibrotic–nitrosative activation, suggesting a potential non-mechanical component of stroke susceptibility.

## 1. Introduction

Patent foramen ovale (PFO) is a common congenital interatrial form of communication, present in approximately 20–25% of the general population, and has been consistently associated with cryptogenic stroke, particularly in younger patients and in individuals without an identifiable alternative cause of stroke [1,2]. Available population-based and stroke cohort analyses suggest that PFO occurs more frequently in patients with embolic stroke of undetermined source than in the general population, supporting its potential causal contribution rather than an incidental finding [3,4]. The prevailing pathophysiological paradigm attributes this relationship primarily to paradoxical embolism, whereby venous thrombi bypass the pulmonary circulation through a right-to-left shunt and enter the systemic arterial circulation [5]. Importantly, the efficiency of this mechanism may depend not only on anatomical patency but also on transient pressure gradients between atria, which can arise during everyday physiological maneuvers and facilitate intermittent right-to-left shunting [6]. This concept is supported by randomized clinical trials demonstrating a reduction in recurrent stroke following transcatheter PFO closure in selected patients [7,8,9]. However, the paradoxical embolism model does not fully explain several clinically relevant observations. These include the occurrence of stroke in patients with small or intermittent shunts, variability in recurrence risk, and the persistence of residual risk even after successful PFO closure [10,11]. In addition, discrepancies between clinical presentation and imaging patterns indicate that a proportion of ischemic events in this setting may arise independently of classical venous embolic sources [12]. Moreover, many individuals with PFO remain asymptomatic throughout life, suggesting that additional biological factors may modulate the clinical expression of this anatomical substrate. This observation points toward interindividual differences in vascular susceptibility, potentially driven by molecular and cellular mechanisms rather than structural characteristics alone [13]. These limitations have prompted increasing interest in non-mechanical mechanisms contributing to stroke susceptibility in patients with PFO. Among these, systemic inflammation and endothelial dysfunction have emerged as key contributors to vascular injury and thromboembolic risk [14,15]. Even subtle, chronic activation of inflammatory pathways may shift endothelial function toward a prothrombotic and proadhesive state, thereby facilitating cerebrovascular events in otherwise low-risk individuals [15,16]. Interleukin-18 (IL-18), a proinflammatory cytokine of the IL-1 family, plays a central role in innate immune activation, endothelial dysfunction, and atherosclerotic plaque destabilization [17,18]. Elevated IL-18 levels have been associated with adverse cardiovascular outcomes and may reflect a proinflammatory vascular phenotype predisposing to thromboembolic events. Experimental and clinical data indicate that IL-18 can enhance endothelial activation and promote coagulation by increasing expression of adhesion molecules and procoagulant factors [17,18]. Galectin-3, a β-galactoside-binding lectin secreted predominantly by activated macrophages, represents a key mediator linking inflammation with fibrosis and tissue remodeling [19,20]. It is involved in extracellular matrix turnover, macrophage activation, and chronic inflammatory signaling, and has been increasingly recognized as a marker of adverse cardiovascular remodeling. Through these mechanisms, galectin-3 may reflect a profibrotic and proinflammatory state not captured by conventional inflammatory markers. In parallel, oxidative and nitrosative stress play a critical role in vascular dysfunction. Reactive oxygen and nitrogen species contribute to endothelial injury, impair nitric oxide bioavailability, and promote prothrombotic conditions [21,22]. Inflammation-driven production of reactive species may further intensify these effects, creating a reinforcing cycle of endothelial impairment [21]. 3-nitrotyrosine (3-NT), a stable product of protein nitration mediated by peroxynitrite, is widely used as a marker of nitrosative stress and has been associated with vascular damage and cardiovascular disease [23,24]. Higher circulating concentrations of 3-NT have been associated with reduced endothelial-dependent vasodilation and increased vascular stiffness, both of which are relevant to stroke pathophysiology [23,24]. Importantly, inflammation, fibrosis-related remodeling, and oxidative/nitrosative stress are not independent processes but form a tightly interconnected biological network. Proinflammatory signaling may induce oxidative and nitrosative stress, while reactive oxygen and nitrogen species further amplify inflammatory and profibrotic pathways through redox-sensitive mechanisms. Taken together, these interactions support the concept of a coordinated biological axis rather than isolated pathogenic pathways [25]. This bidirectional interaction may create a self-perpetuating cycle of vascular injury, forming an integrated inflammatory–fibrotic–nitrosative axis that contributes to endothelial dysfunction and thromboembolic susceptibility [26,27]. Despite increasing recognition of these mechanisms in cardiovascular disease, their role in patients with PFO and cryptogenic stroke remains poorly defined. In particular, it is unclear whether these pathways are activated in a coordinated manner and whether their interaction contributes to stroke risk beyond the classical embolic mechanism. Clarifying these relationships may improve identification of high-risk patients and support the development of therapeutic strategies targeting systemic vascular processes in addition to anatomical correction [28,29]. Therefore, the aim of the present study was to comprehensively characterize inflammatory, fibrotic, and oxidative stress biomarker profiles in patients with PFO and prior cryptogenic stroke in comparison with matched control subjects.

## 2. Materials and Methods

The present study was a prospective, observational, single-center cohort study. The study protocol was approved by the institutional ethics committee, and all procedures were conducted in accordance with the principles of the Declaration of Helsinki and applicable national regulations. Written informed consent was obtained from all participants prior to study enrollment. The study design and reporting were aligned with recommendations for observational studies to ensure methodological transparency and reproducibility.

We prospectively enrolled 92 consecutive patients aged <55 years who underwent percutaneous PFO closure between 2024 and 2025 at the Department of Cardiovascular Diseases, John Paul II Specialist Hospital in Krakow, Poland. All patients in the study group had a confirmed history of cryptogenic ischemic stroke based on comprehensive neurological evaluation and Stroke Unit documentation. The diagnosis of cryptogenic stroke was established following standardized diagnostic algorithms, including neuroimaging and exclusion of large-artery atherosclerosis, small vessel disease, and cardioembolic sources other than PFO. To minimize the potential influence of the post-stroke phase on circulating biomarker levels, patients were enrolled at least 6 months after the cerebrovascular event, which additionally reflected the time required for comprehensive diagnostic evaluation and qualification for transcatheter PFO closure.

Control group consisted of 56 age-matched individuals (<55 years) without PFO and without a history of cerebrovascular events. These participants had previously undergone transesophageal echocardiography (TEE) for clinical indications unrelated to PFO detection, including evaluation of refractory migraine or suspected structural heart disease. The absence of interatrial shunting was confirmed by contrast-enhanced TEE. Control participants were selected to minimize confounding factors related to cardiovascular comorbidities and to ensure comparability with the study population.

Inclusion criteria for the study group were: (1) cryptogenic ischemic stroke occurring before the age of 55 years, confirmed after exclusion of alternative etiologies, and (2) provision of written informed consent. Patients slightly older than 55 years were eligible provided that the index stroke occurred before the age threshold and was confirmed following comprehensive diagnostic work-up. Exclusion criteria included documented supraventricular tachyarrhythmias (including atrial fibrillation), endocrine disorders, known coagulation abnormalities, and pregnancy. Additional exclusion criteria comprised acute or chronic inflammatory diseases, active infection, malignancy, and treatment with immunomodulatory agents, due to their potential influence on circulating biomarker levels.

### 2.1. Transthoracic Echocardiography

Transthoracic echocardiography (TTE) was performed in all participants using a commercially available ultrasound system (Philips EPIQ 7C, Philips Healthcare, Bothell, WA, USA) by an experienced echocardiographer. Image acquisition and measurements were conducted in accordance with current recommendations of the European Society of Cardiology. All examinations were performed using a standardized acquisition protocol to minimize inter-operator variability. A comprehensive echocardiographic examination was undertaken, including detailed assessment of left ventricular dimensions, wall thickness, and global systolic function, as well as evaluation of diastolic function using standard Doppler and tissue Doppler parameters. Left ventricular ejection fraction was determined using the biplane Simpson method whenever image quality allowed reliable assessment. Right ventricular size and systolic performance were assessed using conventional indices. Left atrial size and function were evaluated using standard measurements. Particular attention was directed toward the interatrial septum, with careful assessment for the presence of interatrial communication or shunting. When image quality was suboptimal, additional acoustic windows were employed to optimize visualization of the interatrial septum. All measurements were obtained from standard parasternal and apical views and averaged over multiple cardiac cycles when appropriate to ensure accuracy and reproducibility.

### 2.2. Transesophageal Echocardiography

TEE was performed in all participants using a commercially available ultrasound system (Philips EPIQ 7C, Philips Healthcare, Bothell, WA, USA) by an experienced echocardiographer. All examinations were performed under fasting conditions with continuous monitoring of vital signs. The interatrial septum was systematically examined to determine the presence or absence of a PFO, and PFO morphology, including tunnel length and maximal separation, was assessed using two-dimensional imaging. Measurements were obtained from multiple imaging planes to ensure accurate anatomical assessment. Interatrial shunting was evaluated using both color Doppler and contrast-enhanced imaging techniques. For contrast studies, agitated saline was prepared using 9 mL of saline mixed with 1 mL of the patient’s blood and administered via a peripheral vein. The appearance of microbubbles in the left atrium was assessed at rest and during the Valsalva maneuver. Each examination included analysis of at least three consecutive cardiac cycles and was repeated to ensure consistency and reproducibility of findings. The degree of right-to-left shunting was qualitatively classified as significant or non-significant based on the extent of microbubble passage. In addition to septal assessment, a comprehensive TEE examination included evaluation of pulmonary venous inflow, measurement of pulmonary artery dimensions, and detailed visualization of the left atrial appendage. Particular attention was given to the left atrial appendage, which was carefully examined for thrombus formation and the presence of spontaneous echo contrast in order to exclude alternative cardioembolic sources.

### 2.3. Laboratory Tests

Venous blood samples were obtained from all participants under standardized conditions, following an overnight fast. Blood collection was performed in the morning hours to minimize the influence of circadian variability on biomarker levels. Routine laboratory analyses included fasting glucose, lipid profile, thyroid-stimulating hormone (TSH), renal function parameters (serum creatinine and estimated glomerular filtration rate), N-terminal pro-B-type natriuretic peptide (NT-proBNP), liver enzymes (alanine aminotransferase [ALT] and aspartate aminotransferase [AST]), and complete blood count. These parameters were measured using standard automated laboratory methods in the hospital’s central laboratory. All routine measurements were performed in accordance with internal quality control procedures and laboratory accreditation standards. Circulating levels of IL-18 (detection range 78–5000 pg/mL) and galectin-3 (detection range 0.47–30.0 ng/mL) were determined in serum (both Thermo Fisher Scientific, Vienna, Austria, cat no. BMS267-2 and BMS279-4, respectively) as markers of inflammatory and fibrosis-related activity. Endothelial activation was assessed by measuring intercellular adhesion molecule-1 (ICAM-1; cat no. 201-12-0213, SunRed Biotechnology Company, Wuhan, China; detection range 22–6000 ng/mL) and vascular cell adhesion molecule-1 (VCAM-1; cat no. 201-12-0204, SunRed Biotechnology Company, Wuhan, China; detection range 156–10,000 pg/mL). Plasma concentrations of 3-NT were analyzed as a marker of oxidative/nitrosative stress (Thermo Fisher Scientific; cat no. EEL008; detection range 1.56–100 ng/mL). All ELISAs were commercially available kits, and the measurements were performed according to the manufacturers’ instructions. Laboratory personnel conducting the assays were blinded to the clinical status of the participants to reduce potential measurement bias. The intra- and inter-variability were below 7%.

### 2.4. Statistical Analysis

All data were analyzed using IBM SPSS Statistics 31.0.1 software (IBM Corp., Armonk, NY, USA). The distribution of continuous variables was assessed using the Kolmogorov-Smirnov test. In tables normally distributed variables are presented as mean ± standard deviation (SD), whereas non-normally distributed variables are expressed as median (Q1–Q3). In text normally distributed variables are presented as mean ± standard deviation (SD), whereas non-normally distributed variables are expressed as median, interquartile ranges (Q1–Q3) were additionally included only for the principal analyzed variables in order to preserve overall readability and avoid excessive disruption of the text flow.Categorical variables are presented as counts and percentages. Between-group comparisons were performed using the Student’s *t*-test for normally distributed variables, with homogeneity of variances assessed by Levene’s test, and the Mann–Whitney U test for non-normally distributed variables. Categorical variables were compared using the chi-square test or Fisher’s exact test, as appropriate. Correlation analyses were performed to assess the relationships between the study variables and the concentrations of 3-NT, IL-18, and galectin-3. Depending on the situation, either Pearson’s or Spearman’s correlation coefficients were used. Univariate linear regression analysis was performed for variables significantly associated with 3-NT (galectin-3, IL-18, hsCRP, weight, BMI), followed by multivariable linear regression including variables remaining significant in univariate analysis (IL-18, hsCRP). Additionally, univariate linear regression analysis was performed for variables significantly associated with galectin-3 (IL-18, hsCRP, 3-NT, BMI, ALT, AST), followed by multivariable linear regression including variables remaining significant in univariate analysis (IL-18, hsCRP, AST). Univariate linear regression analysis was performed for variables significantly associated with IL-18 (galectin-3, 3-NT, weight, BMI, hypertension), followed by multivariable linear regression including variables remaining significant in univariate analysis (galectin-3, 3- NT). Variables including age, BMI, hypertension, dyslipidemia, and relevant clinical parameters were initially included in linear and multivariable regression analyses to account for potential confounding effects. Variables that were not statistically significant were not retained in the final multivariable models in order to avoid overfitting and preserve model stability. For clarity and readability, only statistically significant variables were presented in the final regression tables. The models’ effectiveness was assessed using the coefficient of determination (R^2^). A two-tailed *p*-value < 0.05 was considered statistically significant.

## 3. Results

The study cohort included 92 patients with PFO and a documented history of cryptogenic stroke, along with 56 control subjects without PFO or prior cerebrovascular events. The groups were comparable with regard to sex distribution and the majority of anthropometric parameters. Nevertheless, individuals in the PFO group were slightly older (46.0 ± 11.0 vs. 42.5 ± 9.5 years; *p* = 0.048) and had a higher body mass index (25.7 vs. 21.9; *p* = 0.040). Cardiovascular risk factors were more frequently observed among patients with PFO. In particular, the prevalence of hypertension (37.0% vs. 5.4%; *p* < 0.001) and dyslipidemia (76.1% vs. 25.0%; *p* < 0.001) was significantly higher in this group. No significant between-group differences were identified for diabetes mellitus, smoking status, or migraine prevalence (Table 1).

No differences were identified between groups with respect to left ventricular size or systolic and diastolic function. Likewise, atrial dimensions were similar in both groups. Right ventricular systolic performance, evaluated using tricuspid annular plane systolic excursion (TAPSE), was higher in patients with PFO (24.32 ± 3.18 vs. 22.00 ± 3.38 mm; *p* = 0.029), although values in both groups remained within the normal reference range. In the PFO cohort, the mean tunnel length was 10.3 ± 3.6 mm. Septal separation increased significantly during the Valsalva maneuver compared with baseline conditions (median 4.6 mm vs. 2.0 mm; *p* < 0.05) (Table 2).

Patients with PFO displayed a distinct metabolic profile in comparison with controls. Total cholesterol (4.48 vs. 3.96 mmol/L), LDL cholesterol (2.72 ± 0.86 vs. 2.32 ± 0.86 mmol/L), and non-HDL cholesterol (3.08 ± 0.99 vs. 2.66 ± 0.90 mmol/L) were all lower in the PFO group (*p* < 0.01 for all comparisons). In contrast, HDL cholesterol and triglyceride levels were similar between groups. Fasting glucose concentrations did not differ (5.10 vs. 5.20 mmol/L; *p* = 0.513). Liver function parameters were modestly but statistically significantly higher in patients with PFO, with increased alanine aminotransferase (ALT: 27.00 vs. 20.50 U/L; *p* = 0.009) and aspartate aminotransferase (AST: 24.00 vs. 20.50 U/L; *p* < 0.001), although all values remained within reference limits. Renal function, assessed by serum creatinine (73.0 vs. 72.0 µmol/L), as well as thyroid function (TSH: 1.56 vs. 1.63 mIU/L) and NT-proBNP levels (46.0 vs. 39.0 pg/mL), were comparable between groups. A statistically significant difference was observed for estimated glomerular filtration rate (eGFR) (99.00 vs. 95.50 mL/min/1.73 m^2^; *p* = 0.011), although values in both groups remained within the normal range. With regard to inflammatory and endothelial biomarkers, a heterogeneous pattern was observed. High-sensitivity C-reactive protein (hsCRP) levels were lower in patients with PFO compared with controls (0.68 vs. 0.98 mg/L; *p* = 0.049). In contrast, no significant differences were found for white blood cell count (5.94 vs. 6.10 × 10^3^/µL; *p* = 0.591), lipoprotein(a) (9.0 vs. 10.7 mg/dL; *p* = 0.493), VCAM-1 (1310.6 vs. 1213.9 pg/mL; *p* = 0.676), or ICAM-1 (523.7 vs. 621.7 ng/mL; *p* = 0.475). 

**Figure 1:**
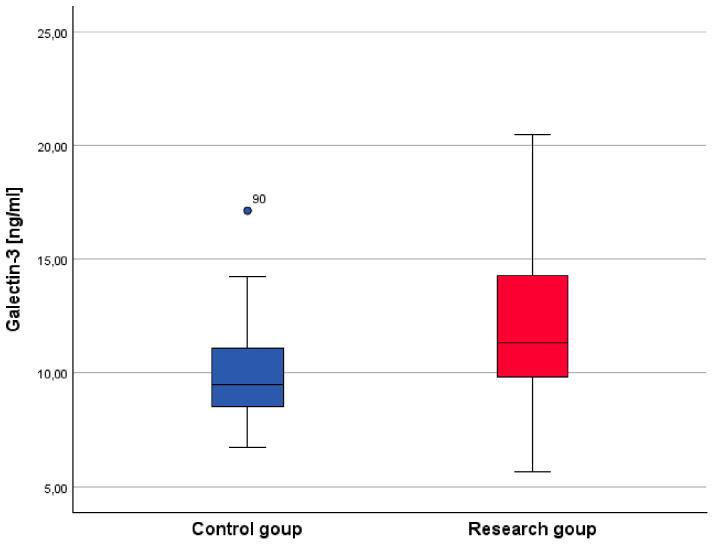
Galectin-3 levels in study groups.

**Figure 2:**
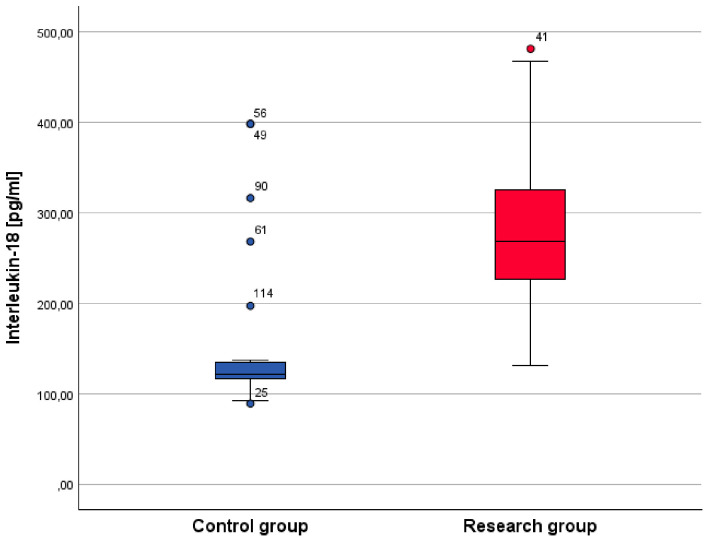
IL-18 levels in study groups.

In contrast to conventional markers, biomarkers reflecting specific inflammatory and remodeling pathways were significantly elevated in the PFO group. Galectin-3 levels were higher in patients with PFO (11.87 [10.00–14.60] vs. 10.36 [8.93–11.27] ng/mL; *p* = 0.015) (Figure 1). The most pronounced difference was observed for interleukin-18 (IL-18), which was markedly increased in the PFO group (268.0 [226.00–325.00] vs. 121.0 [117.00–134.00] pg/mL; *p* < 0.001) (Figure 2) (Table 3).

**Figure 3:**
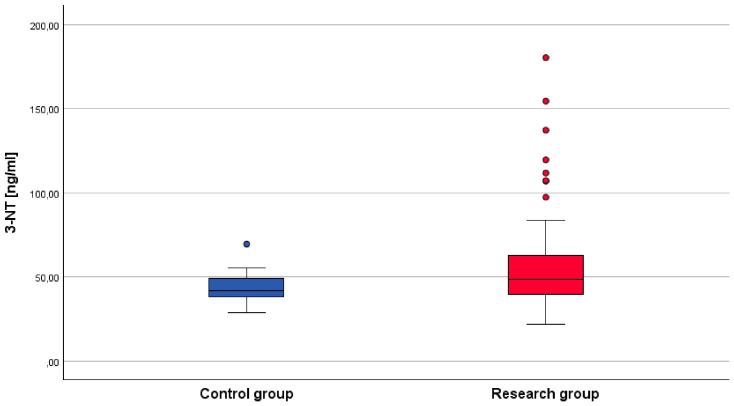
3-NT levels in study groups.

Plasma levels of 3-NT were higher in patients with PFO compared with control subjects (48.5 [39.6–62.5] vs. 41.8 [38.0–49.2] ng/mL; *p* = 0.046), indicating increased oxidative stress in this group (Figure 3).

Correlation analysis revealed significant relationships between IL-18 and galectin-3 (r = 0.565, *p* < 0.001), IL-18 and 3-NT (r = 0.425, *p* < 0.001), and between galectin-3 and 3-NT (r = 0.292, *p* = 0.002), supporting the presence of an interconnected inflammatory–fibrotic–nitrosative axis.

### 3.1. Univariate and Multivariable Linear Regression Analysis of Determinants of 3-NT

In univariate linear regression analysis, galectin-3, IL-18, hsCRP, weight and body mass index were statistically significantly associated with 3-NT (*p* < 0.05). Each 1 ng/mL increase in galectin-3 was associated with a 2.193 ng/mL increase in 3-NT levels (95% CI [0.853; 3.534]), and each 1 pg/mL increase in IL-18 was associated with a 0.114 ng/mL increase in 3-NT levels (95% CI [0.069; 0.159]). Each 1 mg/L increase in hsCRP resulted in a 3.953 ng/mL increase in 3-NT (95% CI [2.578; 5.329]). Each 1 kg increase in weight was associated with a 0.306 ng/mL increase in 3-NT levels (CI [0.0.040; 0.571]) and each 1 kg/m^2^ increase in body mass index was associated with a 0.917 increase in 3-NT levels (CI [0.007; 1.826]).

To identify independent determinants of oxidative stress, variables significant in univariate analysis were entered into a multivariable linear regression model. The coefficient of determination R^2^ was calculated at 0.367, which indicates that the presented model explains 36.7% of the variability in values of 3-NT. The regression coefficient R was 0.616, F 31.476, df 2. No collinearity was observed in the model. Residuals were normally distributed with a mean of approximately zero, homoscedasticity was verified based on the analysis of the scatter plot of residuals against predicted values, no autocorrelation was found (Durbin–Watson 1.529), each of the random components has a normal distribution. In this model, only IL-18 (β = 0.100, *p* < 0.001) and hsCRP (β = 3.648; *p* < 0.001) remained independently associated with 3-NT (Table 4).

### 3.2. Univariate and Multivariable Linear Regression Analysis of Determinants of Galectin-3

In univariate linear regression analysis, IL-18, hsCRP, 3-NT, body mass index, ALT, AST were statistically significantly associated with galectin-3 (*p* < 0.05). Each 1 pg/mL increase in IL-18 was associated with a 0.020 ng/mL increase in galectin-3 levels (95% CI [0.015; 0.026]), and each 1 mg/L increase in hsCRP was associated with a 0.262 ng/mL increase in galectin-3 levels (95% CI [0.055; 0.470]). Each 1 ng/mL increase in 3-NT resulted in a 0.039 ng/mL increase in galectin-3 (95% CI [0.015; 0.063]). Each 1 kg/m^2^ increase in body mass index was associated with a 0.142 ng/mL increase in galectin-3 levels (CI [0.022; 0.261]), each 1 U/L increase in ALT levels was associated with a 0.039 ng/mL increase in galectin-3 levels (CI [0.003; 0.075]) and each 1 U/L increase in in AST levels was associated with a 0.099 ng/mL increase in galectin-3 levels (CI [0.025; 0.174]).

To identify independent determinants of oxidative stress, variables significant in univariate analysis were entered into a multivariable linear regression model. The coefficient of determination R^2^ was calculated at 0.361, which indicates that the presented model explains 36.1% of the variability in values of galectin-3. The regression coefficient R was 0.616, F 20.563, df 3. No collinearity was observed in the model. Residuals were normally distributed with a mean of approximately zero, homoscedasticity was verified based on the analysis of the scatter plot of residuals against predicted values, no autocorrelation was found (Durbin–Watson 1.742), each of the random components has a normal distribution. In this model, only IL-18 (β = 0.018, *p* < 0.001), hsCRP (β = 0.207; *p* = 0.017) and AST (β = 0.072; *p* = 0.026) remained independently associated with galectin-3 (Table 5).

### 3.3. Univariate and Multivariable Linear Regression Analysis of Determinants of IL-18

In univariate linear regression analysis, galectin-3, 3-NT, weight, body mass index and hypertension were statistically significantly associated with IL-18 (*p* < 0.05). Each 1 ng/mL increase in galectin-3 was associated with a 15.820 pg/mL increase in IL-18 levels (95% CI [11.512; 20.128]), and each 1 ng/mL increase in 3-NT was associated with a 1.584 pg/mL increase in IL-18 levels (95% CI [0.955; 2.212]). Each 1 kg increase in weight resulted in a 1.034 pg/mL increase in IL-18 (95% CI [0.058; 2.009]). Each 1 kg/m^2^ increase in body mass index was associated with a 3.902 pg/mL increase in IL-18 levels (CI [0.597; 7.207]). Presence of hypertension was associated with a 48.043 pg/mL increase in IL-18 levels (CI [12.006; 84.080]).

To identify independent determinants of oxidative stress, variables significant in univariate analysis were entered into a multivariable linear regression model. The coefficient of determination R^2^ was calculated at 0.393, which indicates that the presented model explains 39.3% of the variability in values of galectin-3. The regression coefficient R was 0.627, F 36.258, df 2. No collinearity was observed in the model. Residuals were normally distributed with a mean of approximately zero, homoscedasticity was verified based on the analysis of the scatter plot of residuals against predicted values, no autocorrelation was found (Durbin–Watson 1.696), each of the random components has a normal distribution. In this model, only galectin-3 (β = 13.495, *p* < 0.001) and 3-NT (β = 1.060; *p* < 0.001) remained independently associated with IL-18 (Table 6).

## 4. Discussion

The present study demonstrates that patients with PFO and prior cryptogenic stroke exhibit a distinct biological profile characterized by activation of inflammatory, fibrotic and oxidative stress pathways. Importantly, this pattern appears to be selective rather than generalized, suggesting preferential activation of specific molecular pathways rather than a uniform inflammatory response, as described in contemporary cardiovascular research [30]. The key finding of this study is the identification of a coupled inflammatory–fibrotic–nitrosative axis, reflected by elevated levels of IL-18, galectin-3, and 3-NT, as well as their significant interrelationships. This integrated biomarker profile provides additional insight into the systemic component of stroke risk in PFO patients, extending beyond structural cardiac mechanisms previously emphasized in the literature [31]. Importantly, IL-18 levels were markedly higher in patients with PFO and represented the most pronounced difference among all analyzed biomarkers. The extent of IL-18 elevation observed in our cohort supports its potential role as a central mediator within this biological axis and is consistent with prior reports linking IL-18 to vascular inflammation and thrombotic risk [32]. IL-18 is a central mediator of innate immune activation and vascular inflammation, and has been implicated in endothelial dysfunction and atherosclerotic plaque instability [17,18]. The observed elevation of IL-18 in our cohort suggests that patients with PFO may exhibit a proinflammatory vascular phenotype that extends beyond the mechanical paradigm of paradoxical embolism. This observation is in line with evidence indicating that immune-mediated mechanisms may contribute to cerebrovascular risk independently of large-vessel atherosclerosis [33].

In parallel, galectin-3 levels were increased in the PFO group. As a key mediator linking inflammation with fibrosis and tissue remodeling, galectin-3 reflects macrophage activation and extracellular matrix turnover [19,20]. Previous studies have demonstrated that galectin-3 is associated with adverse cardiovascular remodeling and may reflect processes contributing to thromboembolic risk [34]. Importantly, the observed correlation between IL-18 and galectin-3 suggests coordinated activation of inflammatory and profibrotic pathways rather than isolated biomarker abnormalities. Such co-activation has previously been described in cardiovascular conditions characterized by chronic low-grade inflammation, endothelial dysfunction, and progressive tissue remodeling [35]. This relationship may be explained by shared upstream mechanisms, including macrophage-driven signaling and redox-sensitive pathways involved in inflammation-associated fibrosis [35,36].

Moreover, both IL-18 and galectin-3 were significantly associated with 3-NT levels, further supporting the existence of an integrated network linking inflammation, fibrosis, and oxidative stress. These findings support a multidirectional interaction in which oxidative and nitrosative stress amplify inflammatory and profibrotic signaling [37].

Consistent with this interpretation, plasma levels of 3-NT were significantly higher in patients with PFO, indicating increased oxidative/nitrosative stress. Reactive nitrogen species are known to promote endothelial dysfunction, impair nitric oxide signaling, and contribute to a prothrombotic state [21,22,23,24]. The observed association between 3-NT and inflammatory markers suggests that nitrosative stress may act as both a downstream consequence and an amplifier of inflammatory activation. Similar interactions between oxidative stress and inflammatory pathways have been extensively documented in vascular disease models [38].

Interestingly, conventional inflammatory markers such as hsCRP were not elevated in the PFO group and were, in fact, slightly lower compared with controls, although values in both groups remained within normal laboratory reference ranges. This finding suggests that the observed biological alterations may not reflect generalized systemic inflammation but rather selective activation of specific inflammatory pathways. In addition, the control group consisted of individuals referred for transesophageal echocardiography due to clinical indications such as migraine or suspected structural heart disease, which may have influenced baseline inflammatory status. This observation is consistent with previous reports indicating limited sensitivity of hsCRP in detecting pathway-specific or low-grade inflammatory activation [39].

Patients with PFO demonstrated lower total cholesterol, LDL-cholesterol, and non-HDL-cholesterol levels despite a higher prevalence of dyslipidemia. This apparent discrepancy is most likely explained by secondary prevention strategies implemented after the ischemic stroke event. Following cryptogenic stroke, patients are classified as being at very high cardiovascular risk, resulting in lower recommended LDL-cholesterol targets and more frequent diagnosis of dyslipidemia. Furthermore, the lower LDL and non-HDL cholesterol concentrations observed in the PFO group were likely related to more intensive lipid-lowering therapy initiated after stroke and dyslipidemia diagnosis.

The results of regression analyses further support the robustness of these observations. IL-18 remained independently associated with 3-NT levels, while galectin-3 was strongly linked to IL-18. These findings indicate that the relationships between inflammatory, fibrotic, and nitrosative pathways persist even after adjustment for potential confounders, reinforcing the concept of a coupled biological axis. The persistence of these associations in multivariable models is consistent with findings from biomarker-based cardiovascular risk studies [40].

Taken together, these findings provide a potential mechanistic framework extending beyond the traditional embolic model of PFO-associated stroke. While paradoxical embolism remains a key contributor, our results suggest that an underlying state of vascular vulnerability—driven by interconnected inflammatory, fibrotic, and nitrosative processes—may modulate stroke risk. This integrated axis may promote endothelial dysfunction, enhance thrombogenicity, and increase susceptibility to embolic or in situ thrombotic events. This perspective supports a more systemic and integrative understanding of stroke mechanisms in patients with PFO [41].

### 4.1. Clinical Implications

The identification of a coupled inflammatory–fibrotic–nitrosative axis in patients with PFO may have important clinical implications. Importantly, this concept supports a shift from a purely anatomical to a more integrative, biology-driven approach to patient evaluation. First, it suggests that risk stratification in this population could benefit from incorporating biomarker-based assessment in addition to anatomical and clinical factors. Such an approach may allow for more precise identification of patients at increased risk of recurrent cerebrovascular events, particularly among those with low-risk anatomical features. Biomarker profiling may also help differentiate patients in whom systemic vascular vulnerability plays a dominant role from those in whom paradoxical embolism is the primary mechanism. Second, it raises the possibility that therapeutic strategies targeting inflammation or oxidative stress may complement mechanical closure in selected patients. This consideration may be especially important in patients who continue to exhibit an elevated risk profile despite successful PFO closure. Pharmacological modulation of inflammatory or redox pathways could represent a novel adjunctive strategy aimed at reducing thromboembolic susceptibility. Finally, these findings provide a rationale for further studies exploring the role of systemic vascular biology in PFO-associated stroke. Future investigations should assess whether incorporation of biomarker-based strategies translates into improved clinical outcomes and more individualized treatment pathways. In addition, longitudinal studies are warranted to determine whether modulation of this biological axis influences long-term risk of stroke recurrence.

### 4.2. Limitations

Several limitations of this study should be acknowledged. First, this was a single-center study with a relatively modest sample size, which may limit generalizability. Second, the observational design precludes causal inference, and the observed associations should be interpreted as hypothesis-generating. Third, biomarker measurements were performed at a single time point, which does not allow assessment of temporal changes or causality. Another limitation of the present study is the relatively limited biomarker panel. As this was designed as a novel exploratory analysis, representative biomarkers of inflammation, fibrosis, and oxidative stress were intentionally selected to assess the biological plausibility of a coupled inflammatory–fibrotic–nitrosative axis in patients with PFO and cryptogenic stroke. Nevertheless, inclusion of additional cytokines, galectins, and oxidative stress markers could provide a more comprehensive characterization of these pathways and should be addressed in future studies. An additional limitation of the study is that the investigated cohort consisted of patients with both PFO and prior cryptogenic stroke, whereas the control group had neither PFO nor cerebrovascular events. Therefore, the observed biomarker differences may reflect the combined influence of PFO-associated stroke biology, vascular risk burden, and secondary prevention therapy rather than isolated effects of PFO itself. Furthermore, although patient recruitment was performed at least 6 months after the cerebrovascular event to minimize the potential influence of the acute post-stroke phase on circulating biomarker levels, residual effects related to prior stroke cannot be completely excluded. Another limitation of the study is the potential influence of concomitant medical therapy on circulating biomarker levels. Antiplatelet therapy, anticoagulation, lipid-lowering treatment, antihypertensive agents, and other medications may have affected the measured inflammatory, fibrotic, and oxidative stress markers. This issue is particularly relevant given the higher prevalence of dyslipidemia and more intensive secondary prevention strategies in the PFO group. Additionally, although multivariable analyses were performed, residual confounding cannot be excluded. Finally, given the exploratory and hypothesis-generating nature of the study, correction for multiple comparisons was not applied. Therefore, the reported associations should be interpreted cautiously and require validation in larger independent cohorts.

## 5. Conclusions

Patients with PFO and prior cryptogenic stroke exhibit a distinct biological profile characterized by elevated IL-18, galectin-3, and 3-NT levels and their significant interrelationships. These findings support the presence of a coupled inflammatory–fibrotic–nitrosative axis, which may represent an important additional non-mechanical mechanism contributing to stroke susceptibility in this population. This integrated biological framework provides a novel perspective on PFO-associated stroke and may inform future strategies for risk stratification and targeted therapy.

## Figures and Tables

**Table 1:** Baseline Characteristics of the Study Population.

		Control Group (n = 56)	Research Group (n = 92)	*p*-Value
Demographic Characteristics				
Sex [n(%)]	Men	33 (58.9%)	43 (46.7%)	0.150
	Women	23 (41.1%)	49 (53.3%)	
Age [years]		42.5 ± 9.5	46.0 ± 11.0	0.048
Height [cm]		173.0 ± 7.0	170.0 ± 10.0	0.110
Weight [kg]		70.1 ± 14.5	77.1 ± 18.7	0.137
BMI [kg/m^2^]		21.9 (20.8–25.1)	25.7 (22.3–29.9)	0.040
Comorbidities				
Hypertension [n, (%)]		3 (5.4%)	34 (37.0%)	<0.001
Diabetes mellitus type 2 [n, (%)]		1 (1.8%)	3 (3.3%)	0.512
Dyslipidemia [n, (%)]		14 (25.0%)	70 (76.1%)	<0.001
Smoking [n, (%)]		7 (12.5%)	18 (19.6%)	0.266
Migraine [n, (%)]		5 (8.9%)	15 (16.3%)	0.203

BMI—Body Mass Index.

**Table 2:** Echocardiographic Findings in Patients With and Without PFO.

	Control Group (n = 56)	Research Group (n = 92)	*p*-Value
LAA [cm^2^]	33.18 ± 5.30	34.60 ± 5.54	0.182
RAA [cm^2^]	14.00 (11.00–17.00)	15.00 (13.00–17.00)	0.173
RVD1 [mm]	34.00 (31.00–37.00)	35.00 (33.00–38.00)	0.354
LVEDD [mm]	46.23 ± 5.91	46.15 ± 5.63	0.934
LVESD [mm]	30.00 (28.00–33.00)	30.00 (27.00–33.00)	0.737
Interventricular septum—diastole [mm]	9.00 (8.00–10.00)	9.00 (8.00–10.00)	0.762
Posterior wall—diastole [mm]	9.00 (8.00–10.00)	9.00 (8.00–10.00)	0.664
LVEF [%]	65.00 (63.00–65.00)	65.00 (60.00–65.00)	0.489
TAPSE [mm]	22.00 ± 3.38	24.32 ± 3.18	0.029
Ascending aorta [mm]	30.92 ± 5.24	31.15 ± 4.35	0.864
Pulmonary trunk [mm]	20.00 (17.50–21.00)	19.00 (18.00–21.00)	0.631
e’ivs [m/s]	10.00 (9.00–12.00)	10.00 (8.00–12.00)	0.822
e lat [m/s]	12.78 ± 3.52	12.32 ± 4.73	0.782
E/e’	6.28 (5.80–6.70)	6.00 (5.50–7.40)	0.573
E/A	1.20 (0.90–1.30)	1.09 (0.90–1.30)	0.473
RVSP [mmHg]	14.00 (3.00–23.00)	18.00 (5.00–21.00)	0.583
PFO tunnel length [mm]	-	10.3 ± 3.6	-
PFO separation size—rest [mm]	-	2.0 (1.5–2.7)	-
PFO separation size—Valsalva [mm]	-	4.6 (3.7–5.7)	-

LAA—left atrial area; RAA—right atrial area; RVD1—right ventricular dimension 1; LVEDD—left ventricular end-diastolic dimension; LVESD—left ventricular end-systolic dimension; LVEF—left ventricular ejection fraction; TAPSE—tricuspid annular plane systolic excursion; RVSP—right ventricular systolic pressure; PFO—patent foramen ovale.

**Table 3:** Comparison of inflammatory marker levels between patients in the control group and the study group.

	Control Group (n = 56)	Research Group (n = 92)	*p*-Value
hsCRP [mg/L]	0.98 (0.62–2.23)	0.68 (0.37–1.33)	0.049
WBC [10^3^/µL]	6.10 (5.05–7.22)	5.94 (5.25–7.61)	0.591
Lp(a) [mg/dL]	10.7 (7.3–32.35)	9.0 (4.4–17.2)	0.493
VCAM [pg/mL]	1213.9 (890.6–1618.3)	1310.6 (996.7–1609.7)	0.676
ICAM [ng/mL]	621.7 (416.1–762.5)	523.65 (379.3–742.3)	0.475

hsCRP—High-sensitivity C-reactive protein; Lp(a)—Lipoprotein (a); WBC—White blood cells; VCAM—Vascular Cell Adhesion Molecule-1; ICAM—Intercellular Adhesion Molecule-1; IL-18—Interleukin-18.

**Table 4:** Univariate and Multivariable Linear Regression Analysis of Determinants of 3-NT.

	3-NT [ng/mL]			
	B, CI ± 95%	Standard Error B	Standardized Beta Coefficient	*p*-Value
Univariate				
Galectin-3 [ng/mL]	2.193 CI [0.853; 3.534]	0.676	0.292	0.002
IL-18 [pg/mL]	0.114 CI [0.069; 0.159]	0.023	0.425	<0.001
hsCRP [mg/L]	3.953 CI [2.578; 5.329]	0.694	0.488	<0.001
Weight [kg]	0.306 CI [0.040; 0.571]	0.134	0.219	0.025
BMI [kg/m^2^]	0.917 CI [0.007; 1.826]	0.459	0.193	0.048
Multivariable				
IL-18 [pg/mL]	0.100 CI [0.059; 0.141]	0.021	0.378	<0.001
hsCRP [mg/L]	3.648 CI [2.394; 4.902]	0.632	0.450	<0.001

**Table 5:** Univariate and Multivariable Linear Regression Analysis of Determinants of Galectin-3.

	Galectin-3 [ng/mL]			
	B, CI ± 95%	Standard Error B	Standardized Beta Coefficient	*p*-Value
Univariate				
IL-18 [pg/mL]	0.020 CI [0.015; 0.026]	0.003	0.565	<0.001
hsCRP [mg/L]	0.262 CI [0.055; 0.470]	0.105	0.239	0.014
3-NT [ng/mL]	0.039 CI [0.015; 0.063]	0.012	0.292	0.002
BMI [kg/m^2^]	0.142 CI [0.022; 0.261]	0.060	0.226	0.021
ALT [U/L]	0.039 CI [0.003; 0.075]	0.018	0.200	0.036
AST [U/L]	0.099 CI [0.025; 0.174]	0.038	0.247	0.010
Multivariable				
IL-18 [pg/mL]	0.018 CI [0.013; 0.024]	0.003	0.519	<0.001
hsCRP [mg/L]	0.207 CI [0.037; 0.376]	0.085	0.191	0.017
AST [U/L]	0.072 CI [0.009; 0.135]	0.032	0.178	0.026

**Table 6:** Univariate and Multivariable Linear Regression Analysis of Determinants of IL-18.

	IL-18 [pg/mL]			
	B, CI ± 95%	Standard Error B	Standardized Beta Coefficient	*p*-Value
Univariate				
Galectin-3 [ng/mL]	15.820 CI [11.512; 20.128]	2.175	0.565	<0.001
3-NT [ng/mL]	1.584 CI [0.955; 2.212]	0.317	0.425	<0.001
Weight [kg]	1.034 CI [0.058; 2.009]	0.492	0.203	0.038
BMI [kg/m^2^]	3.902 CI [0.597; 7.207]	1.667	0.225	0.021
Hypertension	48.043 CI [12.006; 84.080]	18.190	0.241	0.009
Multivariable				
Galectin-3 [ng/mL]	13.495 CI [9.223; 17.767]	2.156	0.482	<0.001
3-NT [ng/mL]	1.060 CI [0.492; 1.628]	0.287	0.284	<0.001

## Data Availability

The data presented in this study are available on reasonable request from the corresponding author. The data are not publicly available due to privacy and ethical restrictions.

## Abbreviations

The following abbreviations are used in this manuscript:

Q1	first quartile
Q3	third quartile
BMI	Body Mass Index
LAA	left atrial area
RAA	right atrial area
RVD1	right ventricular dimension 1
LVEDD	left ventricular end—diastolic dimension
LVESD	left ventricular end—systolic dimension
LVEF	left ventricular ejection fraction
TAPSE	tricuspid annular plane systolic excursion
RVSP	right ventricular systolic pressure
PFO	patent foramen ovale
hsCRP	High-sensitivity C-reactive protein
Lp(a)	Lipoprotein (a)
WBC	White blood cells
VCAM-1	Vascular Cell Adhesion Molecule-1
ICAM-1	Intercellular Adhesion Molecule-1
Il-18	Interleukin-18
